# Src: Marker or Actor in Prostate Cancer Aggressiveness

**DOI:** 10.3389/fonc.2014.00222

**Published:** 2014-08-18

**Authors:** Virginie Vlaeminck-Guillem, Germain Gillet, Ruth Rimokh

**Affiliations:** ^1^University of Lyon, Cancer Research Centre of Lyon, U1052 INSERM, UMS 3453 CNRS, Lyon I University, Léon Bérard Centre, Lyon, France; ^2^Medical Unit of Molecular Oncology and Transfer, Department of Biochemistry and Molecular Biology, University Hospital of Lyon-Sud, Hospices Civils of Lyon, Lyon, France

**Keywords:** prostate cancer, c-Src, SFK family, tyrosine-kinase, aggressiveness, prognosis, epithelial-to-mesenchymal transition, neuroendocrine differentiation

## Abstract

A key question for urologic practitioners is whether an apparently organ-confined prostate cancer (PCa) is actually aggressive or not. The dilemma is to specifically identify among all prostate tumors the very aggressive high-grade cancers that will become life-threatening by developing extra-prostatic invasion and metastatic potential and the indolent cancers that will never modify a patient’s life expectancy. A choice must be made between several therapeutic options to achieve the optimal personalized management of the disease that causes as little harm as possible to patients. Reliable clinical, biological, or pathological markers that would enable distinctions to be made between aggressive and indolent PCas in routine practice at the time of initial diagnosis are still lacking. The molecular mechanisms that explain why a PCa is aggressive or not are also poorly understood. Among the potential markers and/or actors in PCa aggressiveness, Src and other members of the Src kinase family, are valuable candidates. Activation of Src-dependent intracellular pathways is frequently observed in PCa. Indeed, Src is at the cross-roads of several pathways [including androgen receptor (AR), TGFbeta, Bcl-2, Akt/PTEN or MAPK, and ERK …], and is now known to influence some of the cellular and tissular events that accompany tumor progression: cell proliferation, cell motility, invasion, epithelial-to-mesenchymal transition, resistance to apoptosis, angiogenesis, neuroendocrine differentiation, and metastatic spread. Recent work even suggests that Src could also play a part in PCa initiation in coordination with the AR. The aim of this review is to gather data that explore the links between the Src kinase family and PCa progression and aggressiveness.

## Introduction

Prostate cancer (PCa) is the most common non-skin cancer and the second leading cause of cancer deaths in men from Western countries. Diagnosis can be made fortuitously during the pathological examination of prostate tissue removed because of symptomatic benign prostate hyperplasia, but the disease is more often discovered following prostate biopsies performed because of elevated serum prostate-specific antigen (PSA) levels and/or abnormal digital rectal examination. Another diagnostic scenario is the identification of a PCa at a metastatic stage when metastases (most often bone metastases) become clinically significant. At this time, no curative treatment can be expected and the only therapeutic options are androgen deprivation, which is achieved by surgery (resection of the androgen-secreting testicular tissue), LH–RH analogs (that block testicular androgen secretion), and/or antiandrogens (that block androgen action on the nuclear androgen receptor – AR). Although initially effective, androgen deprivation usually results in an escape with the emergence of a lethal castration-resistant PCa (CRPC). Therapeutic options are more diverse for (apparently) localized, prostate-confined cancers; in this situation, treatments (traditionally radical prostatectomy and radiation therapy) are effective and given with a curative intent. These treatments can, however, have a profound impact on a patient’s quality of life, and clinicians have to be sure that they are provided adequately, in particular, clinicians have to face two interconnected risks (Table 1): (1) the risk of over-treating a localized cancer that will never become life-threatening (the so-called indolent PCa), and (2) the risk to ineffectively treating an aggressive cancer (the so-called high-grade PCa). The pregnant problem when facing a PCa is therefore the need to have an appropriate estimation of its aggressiveness in order to manage the patient with the most appropriate treatment. This estimation is currently based on clinical (organ-confined or not on digital rectal examination), biological (PSA and/or derivatives), and pathological (Gleason score or number/proportion of invaded cores at biopsy) criteria, but remains poorly reliable. Research studies have therefore been performed world-wide to identify and evaluate better criteria for assessing PCa aggressiveness. Efforts are usually focused on molecular markers. The hypothesis is that the better characterization of the intrinsic biological properties of the tumor will lead to more personalized treatment. There are well-established genetic alterations associated with PCa, such as the upregulation of AR-signaling, the overexpression of c-Myc, the loss of the tumor-suppressor gene *Pten* (and subsequent activation of the Akt pathway), and the fusion of *Ets* genes with upstream AR-regulated promoter sequences (with *TMPRSS2-Erg* being the most frequently observed fusion gene). Among others, the c-Src tyrosine kinase (TKs) recently received particular attention because of its implication in several aspects of PCa initiation and progression.

**Table 1:** **The therapeutic stakes of prostate cancer**.

	Life-threatening	Consequences of conventional curative treatments (radical prostatectomy or radiation therapy)	Alternative therapeutic options
Indolent prostate cancer	No	**Over-treatment** (unjustified deterioration of quality of life)	Active surveillance
Intermediate prostate cancer	Yes	**Definitive cure** (justified deterioration of quality of life)	A currently unknown one without deterioration of quality of life
High-grade prostate cancer	Yes	**Ineffective treatment** (incomplete tumor destruction/removal and local or metastatic recurrence)	Androgen deprivation as a unique option or within a (neo-) adjuvant association

**Figure 1:**
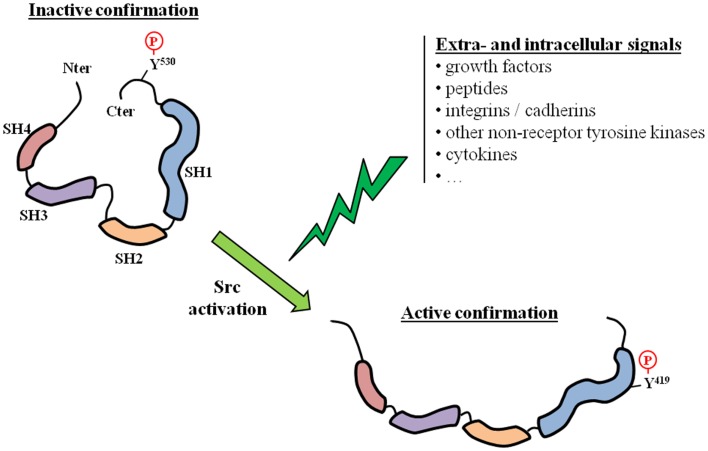
**Src activation**. The activation of Src requires a switch from an inactive to an active conformation. This corresponds to a phosphorylation switch from a tyrosine residue located in the regulatory C-terminal tail (Y530), which is characteristic of the inactive state, to a tyrosine residue located in the catalytic SH1 domain (Y419). Src activation can result from various extra- and/or intracellular signals.

Tyrosine kinases are known to be involved in several fundamental physiological and pathological processes, and include receptor (usually membrane-located) and non-receptor TKs. Src is the prototypical member of the Src family of kinases (SFK), which is the largest family of non-receptor TKs. The SFK family has nine members (Blk, Fgr, Fyn, Hck, Lck, Lyn, Src, Yes, and Yrk) that are implicated in several signal transduction pathways. All SFK members share a similar structure: four Src homology (SH1 to SH4) domains and a unique amino-terminal domain. Src is the most widely studied SFK member, and high resolution crystallographic studies have revealed the complex mechanisms that allow the switch from an inactive to an active state (Figure 1). Src is essentially locked in an inactive conformation through phosphorylation at the tyrosine Y530, which is located in its negative C-terminal regulatory tail. This phosphorylation is performed by the C-terminal Src kinase (Csk) and homologous enzymes. Phosphorylation of tyrosine Y419 in the SH1 kinase domain is the key event associated with protein unfolding and catalytically active conformation. The transition from the off-conformation to the on-conformation occurs upon stimulation from numerous extracellular and intracellular signals. For example, Src and other SFK members are downstream of multiple membrane receptor TKs, including EGFR-Rc and IGF1-Rc. Other interacting membrane proteins are G-protein coupled receptors and integrins. Src is also known to crosstalk with several intracellular compounds such as other non-receptor TKs, steroid receptors, or components of fundamental kinase pathways (PI3K, MAPK, …). For example, Src is usually found within a complex comprising two other non-receptor TKs: focal adhesion kinases (FAK) and Etk. The three kinases are cross-reactive with each other and therefore share the activation process when one of them is activated by a specific stimulus. Overall, Src can be regarded as a scaffolding adaptor between membrane and/or intracellular proteins and these interactions can result in mutual activation/repression depending on phosphorylation exchanges. Src activation has been observed in several cancers, including PCa (1–3), and several lines of evidence link Src and SFKs to prostate carcinogenesis. Src is highly expressed in PCa cell lines (4–9), as well as in the majority of PCa specimens (2, 5, 8, 10), and has become a new therapeutic target. Src inhibitors have recently reached the clinical development stage in managing patients with metastatic PCa.

## Src and Prostate Cancer Initiation

**Figure 2:**
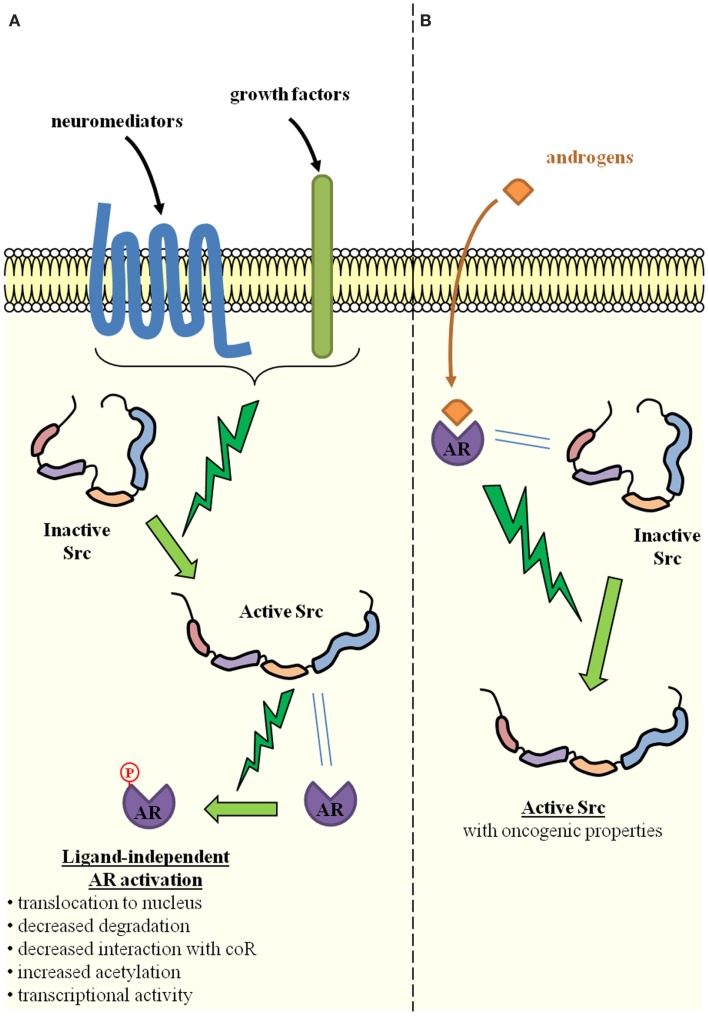
**Reciprocal activation of Src and the androgen receptor through direct physical interaction**. **(A)** Src activation is induced by several extracellular signals such as neuromediators produced by neighboring tumor cells with neuroendocrine differentiation, growth factors produced by neighboring tumor or stromal cells. Through direct physical interaction with the AR, Src is able to phosphorylate the AR and thereby induce ligand-independent AR activation (one of the key mechanisms of castration-resistant prostate cancer). Molecular mechanisms include increased AR translocation to the nucleus, decreased proteasomal degradation, decreased interaction with co-repressors (CoR), and/or increased acetylation. The result is the activation of AR-dependent gene expression programs. **(B)** Conversely, upon ligand binding and direct physical interaction with Src, the AR is able to induce Src activation, which is one of the cellular events associated with oncogenic transformation.

Prostatic intraepithelial neoplasia (PIN) is the putative precursor lesion of PCa. Due to the difficulty in profiling small lesions, little is known about gene expression and genetic alterations that could account for the transition from PIN to cancer. The initiation of PCa has, however, been linked to the loss or mutation of *PTEN* and the subsequent activation of AKT-signaling (11), as well as to *Ets* fusion genes (12). Alterations in AR-signaling have also been advocated, either alone (13) or in combination with the activation of AKT-signaling (14–16). Most of these studies originated from Witte’s laboratory and used an *in vivo* prostate regeneration system in which prostate tissue is regenerated by combining the embryonic urogenital sinus mesenchyme and the postnatal prostate epithelium (12, 14–16). By specifically over-expressing oncogenes of interest, the influence of extrinsic signals on the initiation and progression of PCa can be evaluated. As a result of Src overexpression in many PCa specimens, the overexpression of Src was induced in this system, either alone or in combination with AR overexpression (17). In these experiments, while the overexpression of either Src alone or the AR alone did not significantly change the prostate tubule structure, the simultaneous overexpression of the AR and Src produced sheets of undifferentiated cells with no glandular organization, which is characteristic of a poorly- or un-differentiated PCa. These results are consistent with those previously obtained in the same regeneration system by chronic exposure to paracrine FGF10 (15): induction of PIN and PCa was achieved (15), probably through Src activation, since Src is known to mediate FGF-signaling, while selective Src loss or inactivation inhibited FGF10-induced PIN and PCa (18). A similar effect was observed for Lyn, but not for Fyn, which are other members of the SFK (18). Of interest is the fact that AR overexpression was necessary for the oncogenic potential of wild-type Src, while a constitutively active mutant Src (Y529F) alone phenocopied the synergistic action of the AR and wild-type Src (17). This suggests that the AR is able to activate Src (19, 20). This is a result that is consistent with the detection of increased levels of activated Src in the tumors induced by the simultaneous overexpression of the AR and Src (17). Indeed, cross-activation between the AR and Src is clearly advocated as a way to explain their synergy (17, 21), which is possibly favored by a physical interaction between both proteins (19, 20, 22) (Figure 2). The AR does indeed contain a proline-rich zone that is affine for the Src SH3 domain and allows the formation of an AR–Src complex (19, 20). The oncogenic properties of this complex are also suggested by the influence of the DOC2/DAB2 (differentially expressed in ovarian cancer 2/disabled 2) protein and its partner DAB2IP. *DOC2/DAB2* and *DAB2IP* are considered to be tumor-suppressor genes and are able to counteract the formation and oncogenic action of the AR–Src complex by physically interacting with Src (23, 24). It is notable that the synergy between the AR and ETS-related gene (ERG) has also been suggested as promoting PCa initiation (12). Since functional interactions between the ERG and Src have also been reported (25), whether mutual and possibly synergistic cross-talks between Src, the ERG, and the AR are involved in PCa initiation should be investigated.

## Src and Prostate Cancer Progression

The role of Src and other SFKs in PCa progression has been suggested by a number of demonstrations of their expression in prostate cell lines (4–9) and PCa specimens (5, 8, 10). There are also many reports that SFKs are abnormally activated in PCa cells (1–3), in response to numerous and interconnected stimuli including neuroendocrine ligands (26–28), reactive oxygen species (29), cytokines such as Il-8 (30, 31), growth factors like EGF (27, 29, 32), IGF-1 (28, 33) VEGF (34, 35), or even intracellular activating proteins such as FAK (36). It is notable that all of these molecules are by themselves involved in several basic aspects of cancer progression, including cell proliferation, adhesion, migration, and invasion. SFKs can therefore be regarded as integral components of the signal transduction pathways involved in normal cellular growth, proliferation, migration, and survival, all of which are processes that, if deregulated, promote tumor progression (37–39). With respect to molecular mechanisms, SFKs have been proved to control cell proliferation through the activation of the Ras/ERK/MAPK pathway and to induce specific gene expression programs by affecting the transcriptional activity of several transcription factors like the TGFbeta effectors, the STAT molecules (40). Cell adhesion and migration are also influenced through direct interactions with key partners such as actins, integrins, or kinases such as FAK (40, 41). Indeed, for several years, a great deal of attention has been paid to the roles of Src in these aspects because of the clinical development of Src inhibitors, such as saracatinib (AZD0530), dasatinib, and bosutinib (42). Preclinical *in vitro* studies demonstrated that these inhibitors, along with others that have not yet reached clinical development play a significant role in controlling cell proliferation, adhesion, and migration, as well as the ability to form tumoral xenografts in immunodeficient mice (Table 2). A direct role for Src has not really been demonstrated, probably because the experimental overexpression of Src – without activation – is unlikely to be sufficient in promoting cancer progression. Correlations have in fact been suggested between Src phosphorylation-mediated activation and cell migration (9, 36). Nevertheless, the direct inactivation of Src through siRNA experiments has been proved to induce reduced migration and growth in PCa cell lines (43, 44).

**Table 2:** **Representative studies that evaluated the biological effects of Src inhibitors on basic cellular events associated with prostate cancer progression**.

Reference	Cell lines	Inhibitors	Decreased proliferation	Decreased migration	Decreased adhesion	Apoptosis	Decreased tumoral xenograft growth
(45)	DU-145	PD173955	Yes	–	–	–	–
(36)	DU-145	PP2	–	Yes	–	–	–
	PC-3	
	LNCaP	
(46)	PC-3	CGP77675	Yes	Yes	Yes	No	–
		CGP76080	
(47)	PC-3	Compound 1	Yes	–	–	–	–
(5)	DU-145	Lyn-inhibiting peptides	Yes	–	–	Yes	Yes
	PC-3	
(6)	DU-145	Dasatinib	–	Yes	Yes	–	–
(23)	LNCaP	PP1	Yes (androgen-dependent proliferation)	–	–	–	–
(32)	PC-3	SI35	Yes	Yes	Yes	–	–
		SI40	
(48)	DU-145	Resveratrol	Yes	–	–	Yes	–
(9)	DU-145	Saracatinib	Yes	Yes	–	–	Yes
	PC-3	
(43)	PC-3-MM32GL	Dasatinib	Yes	Yes	–	Yes	Yes (and decreased lymphatic spread)
	PC-3-AR-A1	
	LNCaP	
(49)	DU-145	Bosutinib	Yes	Yes	Yes	–	Yes (and decreased bone metastases)
	PC-3	
(50)	DU-145	Dasatinib	Yes	Yes	–	–	Yes (and decreased angiogenesis)
	PC-3	
	LNCaP	
(44)	CWR22	Dasatinib	–	–	–	–	Yes (androgen-independent growth)
		KX01	
(33)	PC-3	Dasatinib	–	Yes	–	–	–
(51)	PC-3	CTA095	Yes	Yes	–	Yes	Yes

Other studies favor a role of Src in PCa-induced angiogenesis. That Src is a potent actor in angiogenesis has been demonstrated by a substantial amount of evidence. The interplay between Src and VEGF is one of the molecular mechanisms that govern tumor-associated angiogenesis and has also been described with respect to PCa. In prostate cells, hypoxia-induced VEGF expression requires Src activation, which activates Stats3 through phosphorylation and increases HIF1alpha expression (52). Both transcription factors are then able to drive VEGF expression. Il-8 is a known cytokine promoting angiogenesis, and this effect is mediated by Src in prostate cells (31). Src and VEGF cooperation can be targeted by the Src-specific inhibitor dasatinib (50) and several other pharmacological compounds (35, 53–58). Of strong interest for clinical applications, *in vivo* studies have also demonstrated that Src inhibition is able to provoke decreased tumoral xenograft growth by reducing angiogenesis (50, 56).

## Src and Prostate Cancer Metastases

Advanced PCa is frequently associated with metastases. The tumor metastatic cascade includes several steps from the detachment of isolated cells with survival capability from the primary tumor, their migration through the vascular endothelium into the blood stream, their extravasation from the blood stream to the receiving tissue, and the local development of the secondary tumor. To migrate through stromal tissue and invade an adjacent blood vessel, the tumor cell has to develop the ability to survive as an isolated cell, devoid of all contact with other cells and the base membrane. This survival depends on the acquisition of resistance to anoikis, i.e., the variety of apoptosis induced by the loss of the close contacts established between an epithelial cell and the base membrane. Mobility, invasion, and resistance to anoikis are an integral part of a general phenomenon known as the epithelium-to-mesenchyme transition (EMT) (59). EMT is a biological process by which a tumor cell loses some of the features of an epithelial cell (e.g., expression of epithelial markers such as E-cadherin) and acquires some of the characteristics of a mesenchymal cell (expression of mesenchymal markers such as N-cadherin). Through interactions with “true” mesenchymal cells, the modified cell migrates more easily through the stroma. EMT is actually a physiological, evolutionary-conserved process that takes place during normal embryonic and fetal development (gastrulation and morphogenesis) and in some adult situations (wound healing and tissue regeneration) (60, 61). EMT is considered to be the first step of metastatic spread (60), and cells engaged in this process are located at the invasion front (62). Within the metastatic site, a mirror process occurs, whereby the secondary tumor develops like the primary one, including with respect to more or less differentiated epithelial cells (mesenchyme-to-epithelium transition) (63). EMT, or at least EMT-like states, has been described in PCa (59).

An archetypal phenomenon associated with EMT, namely, the so-called cadherin switch from the epithelial E-cadherin to the mesenchymal N-cadherin, is of biological importance as the loss of E-cadherin is associated with the loss of cell-cell junctions. E-cadherin expression is therefore tightly regulated and is notably under the control of EMT-specific transcription factors such as Snail, Slug, Twist, and Zeb1/2. Src activation is known as a potent inducer of EMT and has been proved to induce the dissociation of the complex between E-cadherin and beta-catenin through E-cadherin phosphorylation (39, 64). As a first testimony of Src implication in PCa-associated EMT, decreased E-cadherin expression and increased N-cadherin expression have been linked to Src activation in PCa cells (49, 65–67). Although a detailed and direct demonstration of Src as an inducer of EMT in PCa is still lacking, a few studies support the Src implication by showing a correlation between Src activation and the markers of EMT, such as the mesenchymal aspect of epithelial cells (65), the expression of vimentin (67–69), the action of EMT inducers (70), the expression of EMT-specific transcription factors (65) or micro-RNAs (25, 67), or the EMT-associated increase in cell proliferation, invasion, or mobility (65, 69).

Of note is the fact that EMT is one of the aspects of a larger phenomenon called epithelial plasticity (71). Epithelial plasticity refers to the capacity of cells to undergo reversible phenotypic changes during cell invasion or spread. In other words, the epithelial phenotype is partly lost and replaced by another phenotype, for example a mesenchymal one during EMT. The acquisition of stemness characteristics can be regarded as epithelial plasticity. For PCa, other more specific phenotypes are neuroendocrine differentiation (NED) (72, 73) and osteomimicry (74, 75). These various phenotypes represent the Darwinian adaptation of cancer cells to their environment, are usually transient, and are not mutually exclusive. In a recently published model of EMT induction in PC-3 cells by the inactivation of the E-cadherin gene, expression of stemness, NED, and osteomimicry markers was indeed simultaneously observed (69). While NED is usually induced by androgen deprivation (see below), osteomimicry is a means by which metastatic PCa cells adapt to the bone environment (74–77).

Bones are actually the main metastasis site for PCa cells, which have been shown to deregulate the normal remodeling process that assures bone maintenance. They secrete several molecules such as growth factors and cytokines that alter the tight interplay between osteoclasts and osteoblasts. The balance is in favor of osteoblastogenesis, which explains the usual condensing aspect of PCa-derived bone metastases. Src signaling is a key pathway for bone remodeling regulation, and several lines of evidence link it to the bone metastatic process (78). Several cell models of PCa have linked Src activation with the ability to develop distant metastases (43, 49), while various animal studies also demonstrated the efficiency of Src inhibitors when it comes to reducing bone metastases due to PCa (79–82).

Src therefore seems to be implicated in two crucial steps of the PCa metastatic process: EMT and the consequent cell ability to detach from the primary tumor, and local development of the secondary tumor within the metastatic site. It is also worth noting that the initial arrest and adhesion of cancer cells to the vascular endothelium is also an essential step, being intermediary between the two steps referred to, preceding their extravasation from the blood stream. As well as its role in promoting angiogenesis (see above), Src has also been revealed to play a part in the endothelial permeability that permits cancer cell extravasation (83). In PCa cells, the activated transcription factor Stat5 induces decreased E-cadherin expression as well as the increased adhesion of PCa cells to endothelial cells (84). This effect is inhibited by the Src inhibitor PP2, suggesting that Src also mediates endothelial permeability in PCa (84).

## Src, Neuroendocrine Differentiation, and Resistance to Castration

The development of the embryonic prostate gland and the maintenance of the adult one are very dependent on androgen stimulation. At least during the initial stages of prostate carcinogenesis, PCa cells are naturally androgen-dependent. Androgen deprivation consequently became the first choice treatment for PCas that could not be physically and locally cured. Resistance to castration is the ability of PCa cells to escape and survive, despite androgen deprivation. As the main cause of PCa-related deaths, resistance to castration has been extensively studied and several mechanisms have been advocated such as AR overexpression, AR activation by non-androgenic ligands (because of specific gene mutations that alter the specificity of the ligand binding), or the activation of AR-bypassing signaling pathways (85). Another significant mechanism is AR activation in the absence of any ligand (85). During this process, the AR is subverted through activation by several soluble factors, essential growth factors such as EGF or IGF-1. These factors act by inducing phosphorylation cascades from their TK membrane receptors. As an integrator of several phosphorylation cascade-inducing extracellular signals, Src has logically been advocated as one of the intermediate effectors that transmit the activation signals from growth factors to the AR pathway.

Indeed, a direct physical interaction has been described between Src and the AR (Figure 2). The AR contains a proline-rich region, which has a clear affinity for the Src SH3 domain (19). This interaction is able to relieve Src folding constraints and therefore induces Src activation. Androgens favor the formation of the AR–Src complex, stimulate the Src/Raf-1/Erk-2 pathway, and consequently lead to the increased proliferation of PCa cells (19, 20). Such an interaction is likely to augment the Src oncogenetic effect in prostate cells (17, 18). It should be noted that AR and Src in fact engage in a ternary complex with the estrogen receptor (ER). Complex formation is therefore favored by either androgens or estrogens and conversely inhibited by antiandrogens or antiestrogens. While the AR interacts with the Src SH3 domain, the ER interacts with the SH2 domain (19). Other partners include TK membrane receptors such as IGF1-R that can be overexpressed and activated by AR-stimulated Src (86).

The interaction between the AR and Src not only favors Src activation but also AR activation (Figure 2). It is well known that AR phosphorylation is implicated in AR translocation from the cytoplasm to the nucleus (8), and its transcriptional activity (8, 87–89). A correlation has been demonstrated between AR expression or activity and Src activation in PCa (8, 21, 90). The tyrosine Y^534^ in the AR would be the Src target (17, 19, 21, 91) and its phosphorylation would result in ligand-independent AR activation, which is an important mechanism to explain resistance to castration. Activated Src would also inhibit AR interaction with co-repressors, thereby increasing its capacity to be activated without ligand binding (92).

The AR–Src is a target for several signals that thereby influence Src and/or AR activation. The product of the tumor-suppressor gene DOC2/DAB2 is able to repress PCa cell proliferation through direct interaction with Src and the inhibition of the complex formation (23). The EGF/EGFR pathway activates Src in LNCaP cells that are cultured without androgens, probably through the stimulation of the AR–Src complex formation (93, 94). The use of an antiandrogen does indeed block the proliferative effect, underlying the role of the AR as a downstream effector of the EGF/EGFR pathway (93). Similar actions of several other growth factors are likely since many other receptors are expressed in PCa and are able to interact with and activate Src (95). Finally, the AR gene seems to be able to produce a splice variant, called AR8, which lacks the DNA-binding domain, and is instead located at the plasma membrane and favors interactions between the full-length AR, Src, and EGFR (96).

Resistance to hormone castration is often linked to NED, since NED is almost always the result of androgen deprivation (72, 73). NED is characterized by the production of neuropeptides such as chromogranine A, NSE, serotonin, neurophysin, and synaptophysin. All of these products are thought to exert paracrine effects on adjacent (epithelial) cells, keeping them alive despite androgen deprivation (3). Bombesin is another secreted neuropeptide. Bound to its membrane receptor, it activates Src and thus favors AR phosphorylation and its androgen-independent activation (26, 90). The same action is suggested for other neuropeptides such as neurotensin (26, 27), PTHrp (97), and gastrin-releasing peptide (98). It is, however, still unclear whether these neuropeptides directly activate Src or activate it by stimulating the EGF/EGFR pathway and interactions between Src and EGFR (22, 27, 96, 97). Similar complex interactions could also be observed between the neuropeptides, Src, the AR, and the IGF1-R (28). Another molecular mechanism by which bombesin activates the AR has been suggested: the bombesin is able to stimulate, via Src, p300-mediated AR acetylation (99).

## Src Inhibitors in Clinical Practice

As described above, several experimental studies performed *in cellulo* or in animal models of PCa suggested that molecules able to inhibit Src and/or other members of the SFK could be of clinical interest. Several phase I and phase II clinical trials have therefore been conducted to assess tolerance, side-effects, and optimal dose for three of the several orally bioavailable Src inhibitors: dasatinib (BMSS354825), saracatinib (AZD0530), and KX2-391. The first two act by directly inhibiting the TK activity while the third acts through inhibition of protein–protein interactions. Results were disappointing when saracatinib and KX2-391 were assessed as monotherapy in phase II trials that accrued patients with metastatic CRPC (100, 101). Dasatinib is the most clinically studied SFK inhibitor and has been assessed in several cancers including liquid and solid tumors. It is currently FDA-approved for chronic myelogenous leukemia and, as a second line treatment, for Philadelphia chromosome-positive acute lymphoblastic leukemia. In PCa, dasatinib reached phase II development programs as monotherapy in chemotherapy-naïve men (102, 103), and in men previously treated with one chemotherapy regimen (104), or in association with the cytotoxic docetaxel (105, 106). The relative efficacy of dasatinib (102, 103, 105) and the description of patients with long term survival (104, 106) prompted clinicians to perform phase III trial comparing docetaxel + prednisolone with either dasatinib or placebo (107). Results of this large trial (more than 1500 patients accrued) were disappointing in that the addition of dasatinib did not perform better than docetaxel + prednisolone in terms of overall survival. This failure can be explained by several factors including inadequate study design (108), potential pharmacokinetic interactions between dasatinib and docetaxel (109), a stronger effect on stromal cells than on epithelial cells (despite association with the epithelial-targeted docetaxel), and the too broad specificity of inhibitory effect of dasatinib for numerous receptor and non-receptors TK. Subsequent clinical trials are therefore warranted, which have to be based on an comprehensive knowledge about the optimal timing of a Src inhibitor strategy in PCa progression (before or after chemotherapy? before or after metastatic spreading? …), the intrinsic biology of the patient’s tumor and the value of its association with other new therapeutic agents such as antiangiogenic factors (110).

## Conclusion

Overall, Src has been implicated in several steps of prostate carcinogenesis. It is likely that it plays similar, major roles in other cancers. The particularity of the Src oncogenic action in prostate carcinogenesis is its ability to interfere with the androgen pathway. Through both direct and indirect interaction with the AR, Src is able to reinforce the proliferative and antiapoptotic actions of the AR, even in the absence of specific ligands. These molecular mechanisms constitute a solid rationale in favor of the use of Src inhibitors in routinely managing patients with PCa.

## Conflict of Interest Statement

The authors declare that the research was conducted in the absence of any commercial or financial relationships that could be construed as a potential conflict of interest.
